# Adaptive Extended Kalman Prediction-Based SDN-FANET Segmented Hybrid Routing Scheme

**DOI:** 10.3390/s25051417

**Published:** 2025-02-26

**Authors:** Ke Sun, Mingyong Liu, Chuan Yin, Qian Wang

**Affiliations:** 1 School of Marine Science and Technology, Northwestern Polytechnical University, Xi’an 710072, China; liumingyong@nwpu.edu.cn; 2 Chinese Flight Test Establishment, Xi’an 710089, China; yinc006@avic.com (C.Y.); wangq213@avic.com (Q.W.)

**Keywords:** FANET, SDN, segmented hybrid routing, high dynamic, adaptive extended Kalman prediction

## Abstract

Recently, with the advantages of easy deployment, flexibility, diverse functions, and low cost, flying ad hoc network (FANET) has captured great attention for its huge potential in military and civilian applications, whereas the high-speed movement and limited node energy of unmanned aerial vehicles (UAVs) leads to high dynamic topology and high packet loss rate in FANET. Thus, we introduce the software-defined networking (SDN) architecture into FANET and investigate routing scheme in an SDN-FANET to harvest the advantages of SDN centralized control. Firstly, a FANET segmented routing scheme based on the hybrid SDN architecture is proposed, where inter-segment conducts energy-balanced routing and intra-segment adopts three-dimensional (3D) greedy perimeter stateless routing (GPSR). Specifically, we design the specific process of message interaction between SDN controller and UAV nodes to ensure the execution of the inter-segment routing based on energy balance. Further, to reduce the packet loss rate in high-speed motion scenes, an adaptive extended Kalman prediction algorithm is also proposed to track and predict the 3D movement of UAVs. Simulations verify the effectiveness of the proposed routing scheme in terms of end-to-end delay and packet delivery ratio.

## 1. Introduction

By multiple maneuverable and flexible unmanned aerial vehicles (UAVs) and benefiting from the rapid development of wireless communication technologies, flying ad hoc network (FANET) has attracted considerable attention for its strong survivability, multi-task execution capability, and rapid task switching capability [1,2]. At present, FANET has been widely used in both military and civilian domains, such as disaster site inspection and management, search and rescue operations, forest fire detection, traffic and urban monitoring, environmental sensing, etc. [3,4].

Evolving from mobile ad hoc network (MANET), FANET not only inherits the characteristics of traditional MANET, such as running in a wireless and self-organizing manner, but also has features in dynamic topology change [5,6]. In particular, nodes in FANET move in a three-dimensional (3D) space at high speed and have limited batteries, which makes power consumption a significant concern [7,8]. These characteristics make FANETs frequently suffer from link interruption and data packet loss, which bring challenges to traditional communication mechanisms, especially routing mechanisms [9].

Currently, most of the FANET routing schemes proposed by researchers are based on the modification of routing technology in MANET and vehicular ad hoc network (VANET), because of their similarities [10]. According to the adopted technique, FANET routing algorithms can be divided into topology-based routing algorithms and geographic routing algorithms. The former routing algorithms, such as ad hoc on-demand distance vector routing (AODV) algorithm and destination-sequenced distance vector routing (DSDV) algorithm, generally take up a lot of storage space and consume a significant amount of control overhead to maintain the routing table [11], while only location information is required to conduct greedy routing in geographic routing algorithms [12]. Therefore, extending geographic routing algorithms to 3D space is more suitable for FANET [13,14]. However, 3D geographic routing, such as 3D greedy perimeter stateless routing (3DGPSR), has the problem of 3D void node due to the limitation of the distributed perspective. Although researchers in [15,16,17] proposed some solutions to deal with void node problem in 3D space from the perspective of complex graph theory or flooding, the corresponding cost is larger overhead or worse routing performance.

In order to achieve more reliable and flexible routing, software-defined networking (SDN) is proposed to apply to FANET. By separating the control plane and the forwarding plane, SDN uses a global view of the data flow in network and thus provides a more flexible way to control the FANET routing [18,19]. The authors in [20,21] discussed different SDN-based architectures and the deployment of SDN controllers in UAV networks.

Currently, the centralized SDN architecture based on Openflow is commonly used in FANET. The authors in [22] proposed a routing solution of temporospatial SDN for UAV backhaul network, and they confirmed its advantages in convergence time and network overhead by comparing with distributed approaches, such as AODV, DSDV, and optimized link state routing (OLSR). Considering the power limit of UAVs, the authors in [23,24] focused on the SDN load balancing routing algorithm to maintain a desirable UAV network service. For providing security features through the use of the centralized SDN architecture, the authors in [25] introduced a routing solution for a swarm of cooperative UAVs. The authors in [26] used a modified simulated annealing algorithm to dense FANET environments based on SDN, which considered the temporal information of UAVs to provide an effective routing path. With trajectory tracking information obtained by the SDN controller, the authors in [27] proposed a multipath routing protocol to increase the probability that at least one of the routes is usable. Although the centralized SDN architecture can improve routing reliability exploiting global FANET information obtained through the SDN controller, it requires a lot of control overhead, which will occupy too much wireless bandwidth and affect network expansion of FANET.

To solve this problem, the authors in [28,29] introduced a hybrid SDN architecture and proposed that the mature traditional network can be combined with new benefits of SDN by using the idea of segmented routing (SR). In this regard, the authors in [30,31] applied the SR-based hybrid SDN architecture to MANET and multihop-wireless sensor network, respectively. In these two schemes, the SDN controller only needs to broadcast the list of segment labels to nodes, and then the nodes can make actual forwarding decisions for each segment in a distributed manner, e.g., by running an existing MANET protocol like OLSR. The schemes in [30,31] proved that taking advantage of the global view of SDN, SR can be perfectly implanted; at the same time, with the idea of SR, SDN does not need fine-grained control of all nodes. However, these schemes cannot be applied directly to FANET to deal with high dynamic topology changes. As far as we know, the routing scheme based on the hybrid SDN architecture for FANET has not been studied, which is exactly the motivation of this article.

In addition to the routing framework, position information deviation of UAVs caused by their high maneuverability is also an important factor for FANET routing performance. In [32], authors allowed packets to carry the position and speed message of UAVs, so that UAVs can predict the locations of neighbor nodes and send data directionally. The authors in [33] exploited a geographic position mobility-oriented routing which used a Gaussian Markov model to predict the distance between UAV nodes. Similarly, the authors in [34] proposed a weighted linear regression-based mobility prediction algorithm to predict UAV locations under the adaptive beacon-based GPSR routing framework. Unfortunately, the aforementioned works may misestimate the velocity and the acceleration, since all of them are designed based on a simplified motion model of FANET. Thus, the low precision prediction is not suitable for highly dynamic FANET. With the advantages of low complexity and low cumulative error [35], Kalman algorithm-based prediction algorithm was investigated in [36,37] to predict the trajectory of UAVs. Whereas, only a two-dimensional motion model of UAVs was considered, resulting in inconsistency between algorithm model and practical application scenarios. Thus, it is imperative to investigate a more suitable prediction algorithm to further enhance the accuracy of location information.

Comprehensively considering the advantages of SDN centralized control and the characteristics of FANET, this paper studies a FANET architecture based on the hybrid SDN. On this basis, we propose a segmented routing based on adaptive extended Kalman filter (A-EKF) to adapt to FANET with the characteristics of wireless self-organization, limited energy, and high dynamics. The main contributions are summarized as follows:

1. Taking into account both energy consumption and forwarding distance, this article proposes a segmented routing for FANET, which inter-segment uses energy-balanced routing and intra-segment adopts 3DGPSR. In particular, we design the specific process of message interaction between SDN controller and UAV nodes to ensure the execution of the inter-segment routing based on energy balance.

2. Aiming at the problem of high packet loss rate caused by the high maneuverability of UAVs, this article introduces the A-EKF to track and predict the non-liner 3D movement of UAVs. Specifically, the first-order Taylor expansion is utilized to linearize the nonlinear equation directly. Further, to capture the appropriate parameters without manual adjustment, we also make adaptive improvements on the extended Kalman filter based on the idea of Sage–Husa adaptive Kalman filtering. In this way, SDN controller can obtain more accurate position information of UAVs to improve the timeliness of routing decisions.

3. In order to verify the performance of the scheme proposed in this article, we build a SDN-FANET simulation platform based on OPNET software 14.5 and conduct a series of simulation experiments. Simulation results show that the proposed SDN-FANET segmented hybrid routing based on A-EKF can achieve better performance in FANET.

The rest of this paper is organized as follows. System model is presented in Section 2 . Section 3 describe the SDN-FANET segmented hybrid routing based on A-EKF in detail. While UAV trajectory tracking and prediction algorithm based on A-EKF are explained in Section 4. Simulation model construction and results analysis based on OPNET are given in Section 5. Finally, we conclude this paper in Section 6.

## 2. System Model

### 2.1. Hybrid SDN Architecture for FANET

**Figure 1:**
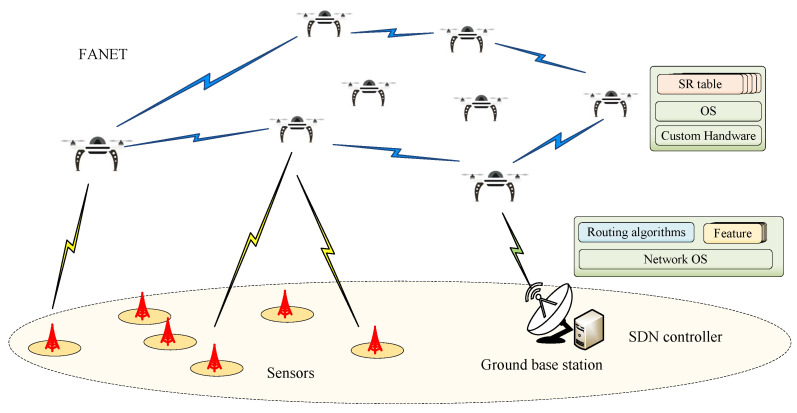
The system model of FANET.

As shown in Figure 1, an Internet of Things (IoT)-applications-oriented FANET is considered in this paper. In the considered FANET, UAV nodes first collect data transmitted by the sensors on the ground. Then, UAV nodes forward the collected data to the gateway according to the multihop path, which is determined by the SDN controller. Finally, the gateway transmits the aggregated data to the ground base station. It is worthwhile to note that UAVs in this system are highly maneuverable in 3D space and run a high-performance routing algorithm based on hybrid SDN architecture. This section first introduces why we adopt the hybrid SDN architecture for FANET.

Traditional FANET applications mostly use distributed control architecture, which is similar to the architecture of MANET. In this architecture, each UAV equipped with an independent operating system can be both a terminal and a router, so that this network has strong scalability and survivability. However, the shortcomings of this architecture are also obvious. On the one hand, the routing paths in FANET will frequently change as the UAVs join, leave, and change position at any time, which makes the limited wireless bandwidth wasted on the discovery and maintenance of the network topology. On the other hand, each node can only obtain local information of the network topology; therefore, the routing decisions made according to this are easily disturbed by the incompleteness and hysteresis of the topology information.

In order to solve the problems in the distributed FANET control architecture, many studies have proposed the centralized SDN control architecture for FANET. In this architecture, UAVs work as white box switches based on OpenFlow in wired networks. They do not have to execute complex routing calculations and only need to perform simple matching operations based on the flow tables issued by the SDN controller. The advantages of this architecture are that it can save computing resources of UAVs and the SDN controller can implement fine-grained control of all data flows based on the global view of FANET. However, for each data flow, the SDN controller needs to configure the flow tables of all nodes on its forwarding path. Especially when the routing path is changed, a high overhead will be generated between the controller and the nodes, which also leads to poor scalability of the network.

Therefore, this article uses a hybrid SDN control architecture based on segment routing as shown in Figure 1. In this architecture, UAVs accept the control of the SDN controller while retaining their own control plane and can perform distributed routing calculations. Specifically, based on global information, the SDN controller does only coarse-grained inter-segment path calculations for data flows according to a certain routing strategy, while fine-grained intra-segment data forwarding paths are calculated by the UAVs. This hybrid SDN control architecture can ease the burden on the controller, reduce bandwidth overhead, and increase the flexibility and reliability of forwarding. Therefore, it is considered an extremely attractive research solution for FANET.

### 2.2. UAV Maneuvering Motion Model

In order to make the routing algorithm well-adapted to the highly maneuverable movement of the UAV in the system model studied in this paper, we add the UAV trajectory prediction mechanism based on A-EKF. However, in order to obtain a more accurate motion prediction result, it is first necessary to establish a suitable UAV maneuvering motion model. For example, if the UAV is moving at a constant speed or at a constant acceleration, it should use the constant velocity model or the constant acceleration model. However, the acceleration of the UAV is generally not constant in the real environment of FANET; that is, the UAV does a highly maneuverable movement, and this kind of maneuvering movement also has some restrictions and regularities. Therefore, this article uses the current statistical model(CSM) to describe the maneuvering movement of the UAVs.

The idea of the CSM is that when the target UAV is moving at a certain acceleration, the acceleration value at the next moment is limited and can only be in the neighborhood of the “current” acceleration. The establishment of this model is based on the following two assumptions. The first assumption is that the mean value of the target acceleration at time *t* ${\bar{a}}_{t}$ is a non-zero value and is the predicted value of the current acceleration. The second is to assume that this acceleration obeys the first-order autocorrelation random noise process, and the statistical characteristics satisfy the modified Rayleigh distribution.

Let $x_{t},{\dot{x}}_{t}$ and ${\ddot{x}}_{t}$ denote the displacement, velocity, and acceleration of the UAV node on x-axis at time *t*, respectively. Then, according to assumption 1, we can get the expression for acceleration as

(1) $${\ddot{x}}_{t}={\bar{a}}_{t}+a_{t},$$

where ${\bar{a}}_{t}$ is a constant in each sampling period, representing the mean value of acceleration, and according to assumption 2, the time-dependent function of $a_{t}$ can be written in exponential form as

(2) $$R_{a}\left(\tau \right)=E\left[a_{t}a_{t+\tau }\right]=\sigma_{a}^{2}e^{-\alpha |\tau |}$$

where $\sigma_{a}^{2}$ is the maneuvering acceleration variance and α is the maneuvering frequency, i.e., the reciprocal of the maneuvering time constant, and its value is generally based on experience. These two parameters represent the mobility characteristics of the node at [t,t+τ]. Based on this formula, it can be further derived that

(3) $${\dot{a}}_{t}=-\alpha a_{t}+w_{t}$$

where $w_{t}$ is Gaussian white noise with a mean value of zero and a variance of $\sigma_{w}^{2}$$\left(\sigma_{w}^{2}=2\alpha \sigma_{a}^{2}\right)$. From Equations (1) and (3), it can be further derived that

(4) $${\dddot{x}}_{t}=-\alpha {\ddot{x}}_{t}+\alpha {\bar{a}}_{t}+w_{t}$$

Finally, we can build the continuous system model of the CSM as

(5) $$\left[\begin{array}{c} {\dot{x}}_{t}\\ {\ddot{x}}_{t}\\ {\dddot{x}}_{t} \end{array}\right]=\left[\begin{array}{ccc} 0 & 1 & 0\\ 0 & 0 & 1\\ 0 & 0 & -\alpha \end{array}\right]\left[\begin{array}{c} x_{t}\\ {\dot{x}}_{t}\\ {\ddot{x}}_{t} \end{array}\right]+\left[\begin{array}{c} 0\\ 0\\ \alpha \end{array}\right]{\bar{a}}_{t}+\left[\begin{array}{c} 0\\ 0\\ 1 \end{array}\right]w_{t}$$

In addition, letting $a_{M}>0$ and $a_{-M}<0$ respectively represent the maximum and minimum values of node acceleration that have been observed, we can get the relationship between the mean value and the variance of acceleration according to the characteristics of the modified Rayleigh distribution. When the node “current” acceleration is positive,

(6) $$\sigma_{a}^{2}=\frac{4-\pi }{\pi }{\left(a_{M}-\bar{a}\right)}^{2},$$

and when the node “current” acceleration is negative,

(7) $$\sigma_{a}^{2}=\frac{4-\pi }{\pi }{\left(\bar{a}-a_{-M}\right)}^{2}.$$

While, when the node “current” acceleration is zero, $\sigma_{a}^{2}$ can be any small positive number.

Similar to the analysis in the *x*-axis direction, we can obtain the expressions of velocity and position in the *y*-axis and *z*-axis directions.

## 3. SDN-FANET Segmented Hybrid Routing Based on A-EKF

**Figure 2:**
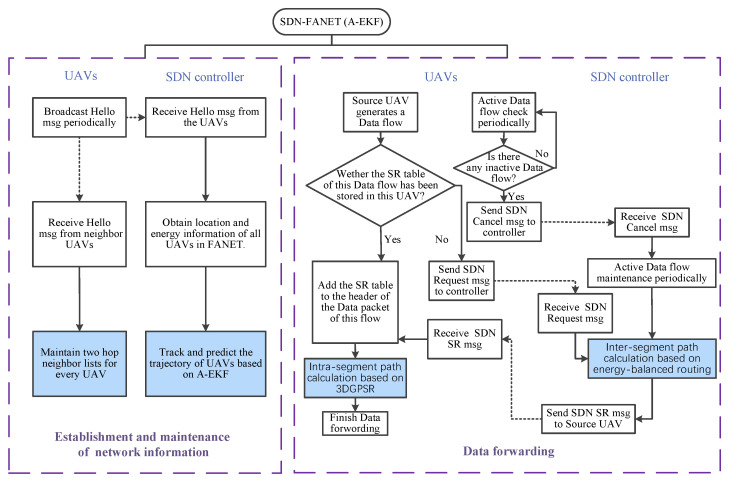
SDN-FANET segmented hybrid routing design based on A-EKF.

In order to improve the communication reliability for FANET, under the hybrid SDN architecture we further study the routing scheme. The detailed design of the proposed SDN-FANET segmented hybrid routing based on A-EKF is presented in Figure 2. This routing scheme mainly consists of two parts. One part is the establishment and maintenance of FANET topology, and the other part is the data forwarding. Next, we further introduce the working mechanism of these two parts specifically.

### 3.1. Establishment and Maintenance of FANET Information

The establishment and maintenance of the topology is the basis of data forwarding and plays a vital role in both the traditional FANET routing mechanism and the SDN-based routing mechanism. In traditional distributed routing algorithms of FANET, UAVs need to send control packets to help exchanging network information. For example, Hello packets are periodically exchanged in AODV, OLSR, and GPSR, etc., to realize neighbor nodes discovery, and MPR selector control packets also need to be flooded in OLSR to maintain the relay link. While in traditional wired SDN network, the controller and switches need to exchange network information through the southbound protocol OpenFlow. Specifically, the controller and switches first exchange Hello packets to establish OpenFlow secure tunnel based on the TCP connection, and then they exchange some control packets based on Link Layer Discovery Protocol (LLDP) to enable SDN controller to obtain the topology information of the entire network. In the SDN-FANET system model proposed in this paper, not only UAVs need to share network information with each other but SDN controller also needs to collect this information from the UAVs. Obviously, if these two control mechanisms in distributed FANET routing algorithms and traditional wired SDN networks are both used to establish and maintain the network information, it will cause a waste of wireless bandwidth resources and computing resources. Therefore, this paper designs a unified control mechanism for the entire SDN-FANET system based on the OLSR control plane.

**Figure 3:**
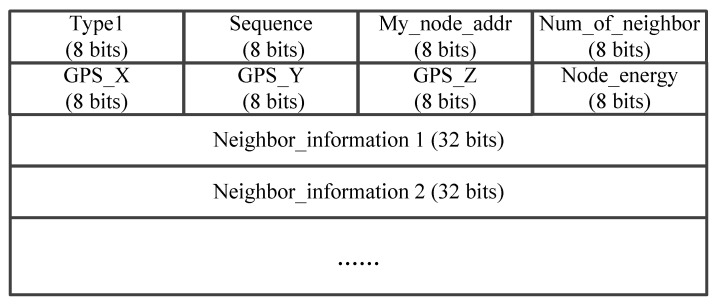
The packet format of Hello.

In SDN-FANET segmented hybrid routing based on A-EKF, the UAV nodes periodically broadcast Hello packets with the frame format, as shown in Figure 3. It can be seen from this figure that the Hello packet not only carries the location and remaining energy information of this UAV, but also the one-hop neighbor nodes information of this UAV. Then, these Hello packets will be received by the one-hop neighbor nodes of this UAV and the SDN controller at the same time. For UAVs, this information can be used to maintain and update the one-hop neighbor list and two-hop neighbor list, so that the distributed UAV nodes can obtain a larger view of the network topology. As for SDN controller, by accepting these Hello messages, it can obtain a global view of the FANET topology without adding additional network burdens, thereby providing a basis for the controller to perform path calculation and data flow control.

**Figure 4:**
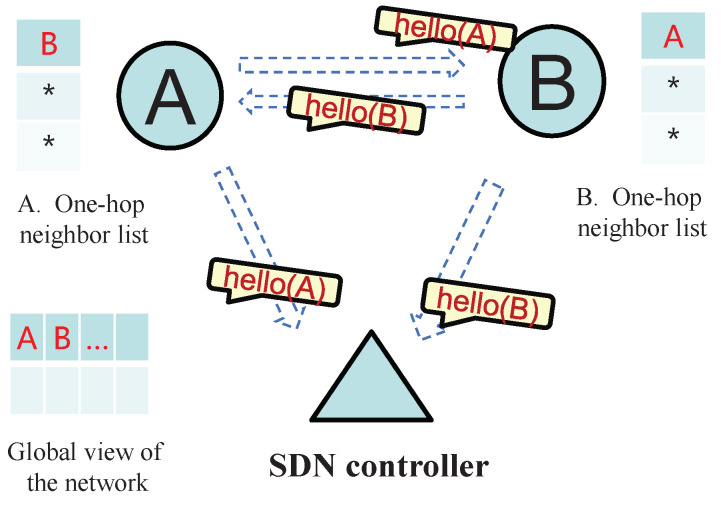
The first round of Hello packet exchange in the SDN-FANET. * indicates the initial state in the neighbor list, i.e., there is no neighbor information yet.

**Figure 5:**
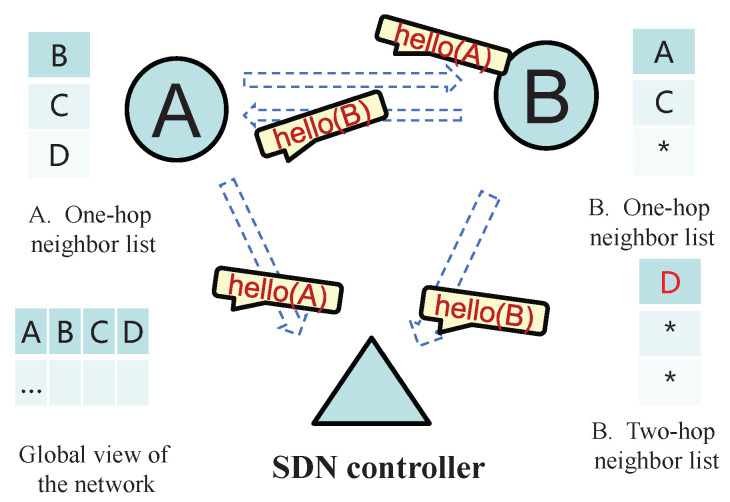
The second round of Hello packet exchange in the SDN-FANET. * indicates the initial state in the neighbor list, i.e., there is no neighbor information yet.

Figure 4 and Figure 5 specifically show the process of establishment and maintenance of network information in the SDN-FANET hybrid routing mechanism. First of all, in the network initialization stage, each UAV writes its own position and energy information into the Hello packet and sends to the SDN controller and its one-hop neighbors. For example, in Figure 4, when neighbor nodes A and B execute the first Hello packet exchange, they will record each other’s information in their one-hop neighbor lists, and SDN controller will also obtain the information of nodes A and B. Next, before the next Hello packet broadcast, the UAV nodes also need to write their one-hop neighbors’ information into the Hello packets. As shown in Figure 5, we suppose that before nodes A and B exchange Hello for the second time, node A discovers its one-hop neighbors C and D, while node B discovers its one-hop neighbor C. Then, when A and B exchange Hello packets for the second time, node B can find its own two-hop neighbor node D by comparing its own one-hop neighbor list with node A’s one-hop neighbor list. While in this process, SDN controller and node A will only update the location and energy information of the related nodes because they cannot find new nodes in the process of parsing the Hello packets.

Through the above method, as long as the UAV nodes in the network execute two rounds of Hello packet exchange, all the UAV nodes can obtain all their neighbors’ information within two hops, and the SDN controller also can obtain the information of all UAV nodes in the FANET. However, due to the high-speed flying of the UAVs, when the position information is sent to the SDN controller, it is usually outdated. Therefore the SDN controller will further process the obtained position information of these UAV nodes based on the A-EKF algorithm, so as to realize the tracking and prediction of the movement of the UAVs and provide a more accurate routing path calculation basis. The mechanism of UAV trajectory tracking and prediction based on A-EKF will be described in detail in Section 4.

### 3.2. Data Forwarding Based on Segmented Source Routing

**Figure 6:**
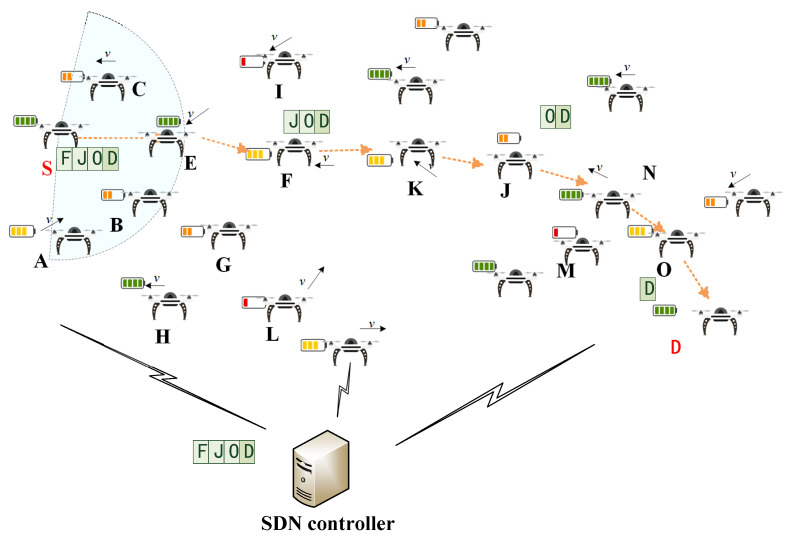
Data forwarding based on segmented source routing in FANET.

**Figure 7:**
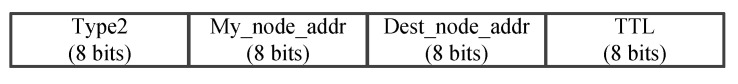
The packet format of SDN_request.

**Figure 8:**
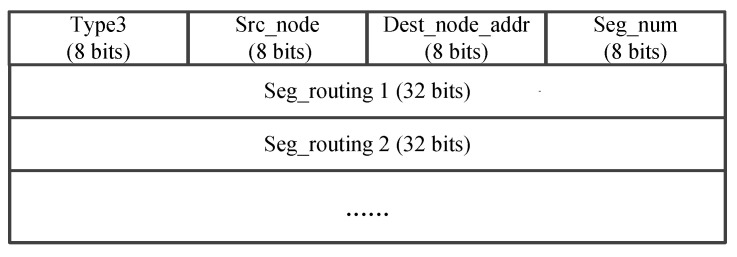
The packet format of SDN_SR list.

**Figure 9:**
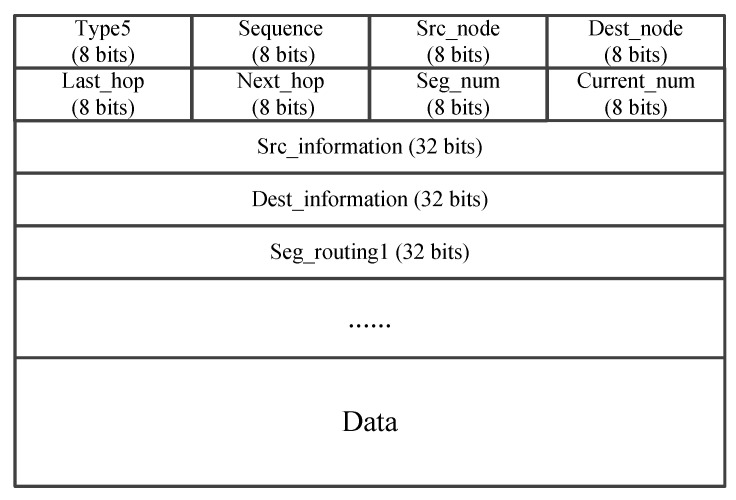
The packet format of SDN_data.

**Figure 10:**
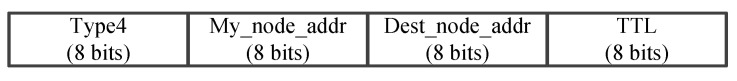
The packet format of SDN_cancel.

The process of the data forwarding based on segmented source routing in FANET as shown in Figure 6. In this figure, we assume that there is a data flow from the source node S to the destination node D after the initialization is complete. At this time, the source node S first needs to send the SDN_request packet in the frame format as Figure 7 to the SDN controller, which includes the source node ID, the destination node ID, and the Time To Live (TTL) of this request message. When the SDN controller receives this message, it will use the energy-balanced routing to calculate the inter-segment path. If the calculated shortest path passes through the nodes S, E, F, K, J, N, O, and D, the controller will send the SDN_SR list to the source node S as Figure 8, which contains the results of F, J, O, and D in the Seg_routing fields. That is, the key nodes of the inter-segment path are selected at intervals of two hops. After receiving the SDN_SR list of this data flow, the source node S caches the result locally and fills it in the header of the SDN_DATA packet as shown in Figure 9. Then, according to the two-hop neighbor list, the source node S can easily find the intra-segment path using the 3DGPSR algorithm, i.e., the data can be forwarded to F through node E. When node F receives this data packet, it will find the path to the segment destination node J and continue forwarding. The above forwarding process will be repeated until the data reaches the destination node D. The important thing is that if there is a data flow from S to D again, it no longer need to send an SDN_request to the SDN controller, but only needs to retrieve the locally cached SDN_SR list and fill it in the header of the SDN_DATA packet, which greatly reduces the routing delay. In addition, it is worth noting that changes in the position and energy of UAV nodes will affect the results of the SDN_SR list. Therefore, the SDN controller needs to continuously monitor the active data flows and periodically calculate their SDN_SR list. If the results change, SDN controller should actively send the new SDN_SR list to the source nodes. If a source node has not initiated a data flow to a destination node for a long time, i.e., this data flow is already inactive, the source node will send the SDN_cancel packet as shown in Figure 10 to the SDN controller to notify the SDN controller that it no longer needs to monitor this data flow.

Next, we respectively introduce the calculating methods of inter-segment path and intra-segment path.

#### 3.2.1. Inter-Segment Path Calculation

The calculation of the inter-segment routing on the SDN controller adopts the routing based on energy balance, i.e., we comprehensively consider the two indicators of distance and energy in the weighted Dijkstra algorithm. First of all, the energy information in the Hello packets should be the energy level which has been discretized. Because if the continuously changing energy is used directly, the results of inter-segment routing may change frequently, which increases the network overhead. Let $E_{0}$ represent the initial energy of a UAV, $Q_{0}$ represent its initial energy level, and $E_{\mathrm{remain}}$ represent its remaining energy, then its current energy level $Q_{\mathrm{cur}}$ can be expressed as

(8) $$Q_{\mathrm{cur}}=\left\lceil \frac{E_{\mathrm{remain}}}{E_{0}}\times Q_{0}\right\rceil .$$

Then, after obtaining the location and energy level of all UAV nodes through the Hello packets, SDN controller can build a complete network topology $G=\left(V,L\right)$, where *V* represents the set of UAV vertices, and *L* represents the set of directed edges. Assuming that data is sent from node *i* to its neighbor node *j*, the weight $u_{ij}$ of the directed edge $L_{ij}$ can be expressed as

(9) $$u_{ij}=\exp\left(\frac{Q_{0}}{q_{j}}-1\right)\times d_{ij},$$

where $q_{j}$ is the current energy level of receiving node *j* and $d_{ij}$ is the distance between two nodes. There are two points worth explaining. On the one hand, the exponential function is used to enhance the influence of energy level changes on the weight, i.e., when the energy level $q_{j}$ decreases, the weight $u_{ij}$ will increase exponentially. On the other hand, this weight takes into account the energy level of the receiving node *j* but not the energy level of the sending node *i*. This is because, on a forwarding path composed of multiple directed edges, the sending node of the next edge is also the receiving node of the previous edge, and the sending node of the first edge is the source node of a data flow. The energy consumption of the source node cannot be avoided by the choice of the link, so we can ignore the influence of the energy level of the sending node. Finally, based on the above established directed network graph, the inter-segment path result can be obtained by the weighted Dijkstra algorithm.

#### 3.2.2. Intra-Segment Path Calculation

**Figure 11:**
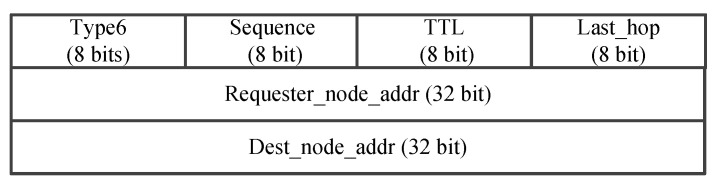
The packet format of RREQ.

**Figure 12:**
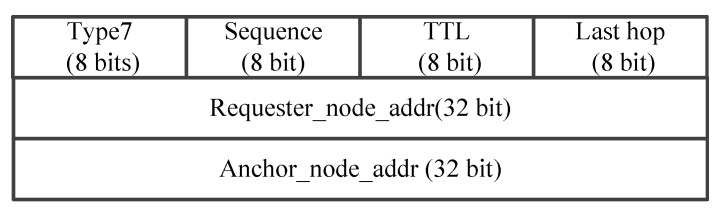
The packet format of RREP.

Intra-segment path calculation based on 3DGPSR. After the UAV nodes receive the SDN_SR list, the calculation of the intra-segment route is carried out using 3DGPSR. Specifically, the UAV node first checks whether the destination node of the segment is its one-hop neighbor. If yes, the data will be sent directly to the destination node of the segment. Otherwise, the data will be forwarded to the neighbor which is closest to the destination node of the segment. However, due to the highly dynamic movement of the UAV in the three-dimensional space, in this greedy forwarding process, node may not be able to find a neighbor closer to the segment destination node, i.e., it may encounter a void node and cannot continue greedy forwarding. At this time, we adopt the method of perimeter forwarding, hoping to find other paths to the destination node through the method of limited flooding. In particular, the route request (RREQ) packets is first created by the current forwarding node, and then the current forwarding node broadcast RREQ to neighbors. Here, the information of the destination node, the requester node, and the TTL of the destination node are carried in the RREQ packet, which can be seen in Figure 11. Further, UAV node can utilize the Last Hop field in RREQ to record the forwarding route. Once one UAV node receives the RREQ, it will look up the neighbor list to determine whether the destination node is available. If the destination node is available, this receiving node can certainly avoid the routing holes, i.e., it is an anchor. Otherwise, this receiving node will continue broadcasting the received RREQ packet to its neighbors, as long as the the TTl of this RREQ packet is non-zero or no anchor node is available. For the anchor node, it will create a route reply (RREP) packet and transmit it to the request node, as shown in Figure 12 . In this way, by assembling 3DGPSR, UAV nodes can not only complete the calculation of inter-segment path, but also ensure the smooth progress of data forwarding when the controller fails or the topology changes rapidly.

## 4. UAV Trajectory Tracking and Prediction Based on A-EKF

To the end, we have obtained the the SDN-FANET segmented hybrid routing scheme based on the adaptive Kalman filter. However, the outdated location information contained in Hello packet can decrease the routing performance. For this problem, we further propose a trajectory tracking and prediction algorithm for the routing scheme in Section 3.

Being a minimum variance estimation-based recursive algorithm, Kalman filter can not only estimate hidden variables from measurement, but also predict future state of a system based on past estimation data. For the standard Kalman filter algorithm, it should be guaranteed that the statistical characteristics of random signals follow Gaussian distribution and the standard Kalman filter algorithm only performs well in linear systems. However, in engineering practice, the system always has uncertain interference, which causes its model to be non-linear. As the CSM described in Section 2, the UAV maneuvering motion model concerned in this paper may have accelerations which can change at anytime, so it is also a nonlinear model. Therefore, we adopt the extended Kalman filter, which basic idea is to use the first-order Taylor expansion to linearize the nonlinear equation directly, and then use the generalized Kalman filter iteration method to perform filter estimation. Moreover, based on the idea of Sage–Husa adaptive Kalman filtering, we make adaptive improvements to the above-mentioned extended Kalman algorithm, so that the model noise parameters can be adaptive adjustment.

First of all, in order to describe the motion state of a UAV, we define the state variable of EKF as

(10) $$X={\left[x,v_{x},a_{x},\varepsilon_{x},y,v_{y},a_{y},\varepsilon_{y},z,v_{z},a_{z},\varepsilon_{z}\right]}^{T}$$

where $x,v_{x},a_{x},y,v_{y},a_{y},z,v_{z},a_{z}$ respectively represent the displacement, velocity, and acceleration of the UAV in the three directions of the 3D rectangular coordinate system, while $\varepsilon_{x},\varepsilon_{y},\varepsilon_{z}$ respectively represent the total position error caused by various error sources in three directions.

Since the total error caused by the various error sources in each direction can be characterized by a current mean and a sum of colored noise conforming to a first-order Markov process, according to the “current” statistical model described in Section 2, the state equation of the system modeled with extended Kalman can be defined as

(11) $${\dot{X}}_{t}=AX_{t}+U_{t}+W_{t}$$

where $U_{t}$ is the mean of “current” acceleration vector and $W_{t}$ is the noise vector. Here, A represents the system state transition matrix, which can be expressed as

(12) $$A=\left[\begin{array}{ccc} A_{x} & 0_{4\times 4} & 0_{4\times 4}\\ 0_{4\times 4} & A_{y} & 0_{4\times 4}\\ 0_{4\times 4} & 0_{4\times 4} & A_{z} \end{array}\right]$$

where

(13) $$A_{x}=\left[\begin{array}{cccc} 0 & 1 & 0 & 0\\ 0 & 0 & 1 & 0\\ 0 & 0 & -1/\tau_{ax} & 0\\ 0 & 0 & 0 & -1/\tau_{x} \end{array}\right]$$

(14) $$A_{y}=\left[\begin{array}{cccc} 0 & 1 & 0 & 0\\ 0 & 0 & 1 & 0\\ 0 & 0 & -1/\tau_{ay} & 0\\ 0 & 0 & 0 & -1/\tau_{y} \end{array}\right]$$

(15) $$A_{z}=\left[\begin{array}{cccc} 0 & 1 & 0 & 0\\ 0 & 0 & 1 & 0\\ 0 & 0 & -1/\tau_{az} & 0\\ 0 & 0 & 0 & -1/\tau_{z} \end{array}\right]$$

Further, $U_{t}$ and the noise vector $W_{t}$ in (11) can be respectively written as

(16) $$U_{t}={\left[0,0,\frac{{\bar{a}}_{x}}{\tau_{ax}},0,0,0,\frac{{\bar{a}}_{y}}{\tau_{ay}},0,0,0,\frac{{\bar{a}}_{z}}{\tau_{az}},0\right]}^{T}$$

and

(17) $$W_{t}={\left[0,0,\omega_{ax},\omega_{x},0,0,\omega_{ay},\omega_{y},0,0,\omega_{az},\omega_{z}\right]}^{T}$$

Here, $\tau_{x},\tau_{y},\tau_{z}$ are time constants of the Markov process, $\tau_{ax},\tau_{ay},\tau_{az}$ are time constants of the acceleration, ${\bar{a}}_{x},{\bar{a}}_{y},{\bar{a}}_{z}$ are mean value of “current” acceleration in the direction of three axes. Moreover, $\omega_{ax},\omega_{ay},\omega_{az}$ are white Gaussian noises with zero mean and variance being as $\sigma_{ax}^{2},\sigma_{ay}^{2},\sigma_{az}^{2}$, respectively. The purpose of the above modeling process is to model the total position error of a UAV as a state variable augmented by colored noise, i.e., a first-order Markov process.

Then, the modeling of measurement equation is introduced as follows. The positioning measurement results in the directions of the three coordinate axes are defined as $Z_{x},Z_{y},Z_{z}$; we have

(18) $$Z_{x}=x+\varepsilon_{x}+\omega_{cx}$$

(19) $$Z_{y}=y+\varepsilon_{y}+\omega_{cy}$$

(20) $$Z_{z}=z+\varepsilon_{z}+\omega_{cz}$$

Thus, the observation equation can be obtained as

(21) Z=HX+V

where $Z={\left[Z_{x},Z_{y},Z_{z}\right]}^{T}$, and the observation matrix H can be expressed as

(22) $$H=\left[\begin{array}{cccccccccccc} 1 & 0 & 0 & 1 & 0 & 0 & 0 & 0 & 0 & 0 & 0 & 0\\ 0 & 0 & 0 & 0 & 1 & 0 & 0 & 1 & 0 & 0 & 0 & 0\\ 0 & 0 & 0 & 0 & 0 & 0 & 0 & 0 & 1 & 0 & 0 & 1 \end{array}\right]$$

Further, the observation noise vector can be written as

(23) $$V={\left[\omega_{cx},\omega_{cy},\omega_{cz}\right]}^{T}$$

To this end, we have initialized the variables according to the above system model and the actual observation results. In the following, we can conduct filtering and prediction according to the following iterative process of the extended Kalman filter algorithm. Firstly, we estimate the current state from the previous state, which can be written as

(24) $${\hat{X}}_{k|k-1}=\Gamma_{k|k-1}{\hat{X}}_{k-1}$$

Secondly, a prior error covariance matrix $P_{k-1}$ can be estimated based on the previous error covariance matrix $P_{k|k-1}$

(25) $$\begin{array}{c} P_{k|k-1}=\Phi_{k|k-1}P_{k-1}{\Phi_{k|k-1}}^{T}+Q_{k-1} \end{array}$$

Thirdly, based on the discretization matrix of the system observation noise covariance matrixas R, we calculate the Kalman gain as follows:

(26) $$K_{k}=P_{k|k-1}H_{k}^{T}{\left({H_{k}}^{T}P_{k|k-1}H_{k}+R_{k}\right)}^{-1}$$

Then, the state estimation result ${\hat{X}}_{k}$ is updated according to the measurement results

(27) $${\hat{X}}_{k}={\hat{X}}_{k|k-1}+K_{k}\left(Z-H_{k}{\hat{X}}_{k|k-1}\right)$$

Finally, the posterior estimated covariance is updated as follows

(28) $$P_{k}=\left(I-K_{k}H_{k}\right)P_{k|k-1}{\left(I-K_{k}H_{k}\right)}^{T}+K_{k}R_{k}K_{k}^{T}.$$

The above parameters in the extended Kalman filter are defined as

(29) $$\Gamma_{k|k-1}=\left[\begin{array}{ccc} \Gamma_{x} & O_{4\times 4} & O_{4\times 4}\\ O_{4\times 4} & \Gamma_{y} & O_{4\times 4}\\ O_{4\times 4} & O_{4\times 4} & \Gamma_{y} \end{array}\right]$$

where

(30) $$\Gamma_{x}=\left[\begin{array}{cccc} 1 & T & \frac{T^{2}}{2} & 0\\ 0 & 1 & T & 0\\ 0 & 0 & 1 & 0\\ 0 & 0 & 0 & e^{-\frac{T}{\tau_{x}}} \end{array}\right]$$

Here, the expressions of $\Gamma_{y}$ and $\Gamma_{z}$ are similar to $\Gamma_{x}$, where *T* denotes the sampling time interval at which the data is filtered.

Moreover, $\Phi_{k|k-1}$ is the discretization matrix of the system state transition matrix A, and we have

(31) $$\Phi_{k|k-1}=\left[\begin{array}{ccc} \Phi_{x} & O_{4\times 4} & O_{4\times 4}\\ O_{4\times 4} & \Phi_{y} & O_{4\times 4}\\ O_{4\times 4} & O_{4\times 4} & \Phi_{y} \end{array}\right]$$

where

(32) $$\Phi_{x}=\left[\begin{array}{cccc} 1 & T & \left(\frac{T}{\tau_{ax}}-1+e^{-\frac{T}{\tau_{ax}}}\right){\tau_{ax}}^{2} & 0\\ 0 & 1 & \left(1-e^{-\frac{T}{\tau_{ax}}}\right)\tau_{ax} & 0\\ 0 & 0 & e^{-\frac{T}{\tau_{ax}}} & 0\\ 0 & 0 & 0 & e^{-\frac{T}{\tau_{x}}} \end{array}\right]$$

Here, the system state transition matrix $\Phi_{y}$ $\Phi_{z}$ are similar to $\Phi_{x}$.

Furthermore, Q is the discretization matrix of the system noise covariance matrix, i.e.,

(33) $$Q=diag[0,0,\sigma_{ax}^{2},\sigma_{x}^{2},0,0,\sigma_{ay}^{2},\sigma_{y}^{2},0,0,\sigma_{az}^{2},\sigma_{z}^{2}]$$

while R is the discretization matrix of the system observation noise covariance matrix, i.e.,

(34) $$R=diag[{R_{x}}^{2},{R_{y}}^{2},{R_{z}}^{2}]$$

According to the current statistical model, we can obtain mean ${\bar{a}}_{x},{\bar{a}}_{y},{\bar{a}}_{z}$ and variance $\sigma_{ax}^{2},\sigma_{ay}^{2},\sigma_{az}^{2}$ of the three coordinate axis directions in the above modeling process. Taking the X-axis direction as an example, it can be determined by the following two equations

(35) $${\bar{a}}_{x}\left(k\right)={\bar{a}}_{x}\left(k|k-1\right)$$

and

(36) $$\sigma_{ax}^{2}=\left\{\begin{array}{c} \frac{4-\pi }{\pi }{\left[a_{xM}-{\bar{a}}_{x}\right]}^{2}\;,\;\;\;\;\;{\bar{a}}_{x}\;\geq 0\;\;\;\\ \frac{4-\pi }{\pi }{\left[{\bar{a}}_{x}-a_{-xM}\right]}^{2},\;\;\;\;\;{\bar{a}}_{x}\;<0 \end{array}\right.$$

At this point, we have completed the modeling of the extended Kalman filter based on the “current” statistical model. However, in practice, the excellent performance of Kalman algorithm depends on both the accurateness of modeling and the estimation accurateness of the noise parameters. Although the above “current” statistical model tries to approximate the noise in a statistical manner, there is still a certain gap with the actual motion noise parameters of UAV. Therefore, in order to improve the performance of tracking and prediction of UAV motion, based on the idea of Sage–Husa adaptive Kalman filter algorithm, we propose an adaptive extended Kalman algorithm model, which can adaptively capture the appropriate parameters without manual adjustment. In the following, we explain how the A-EKF can improve the performance of EKF from two aspects. The first aspect is that we introduce an adaptive forgetting factor, the specific theoretical explanations of which are shown from Equation (37) to Equation (38). The second aspect is that we introduce the adaptive adjustment formula of $Q_{k}$ and $R_{k}$ into the loop iteration process of Kalman filter, the specific theoretical explanations of which are shown from Equation (39) to Equation (41).

Firstly, an adaptive forgetting factor is introduced into (25), which can be rewritten as

(37) $$\begin{array}{c} P_{k|k-1}=\lambda_{k}\Phi_{k|k-1}P_{k-1}{\Phi_{k|k-1}}^{T}+Q_{k-1} \end{array}$$

When the state changes suddenly, the information of old state may result in estimation error of current state and thus reduce the accuracy of perdition. In this case, we should make full use of the newly measured data to improve the performance of the filter in dynamic scenario. Therefore, we introduce the forgetting factor to limit the memory length of the Kalman filter. Here, the optimal forgetting factor can be expressed as $\lambda_{k}=max\left\{1,tr\left(N_{k}\right)/tr\left(M_{k}\right)\right\}$ where $M_{k}=H_{k}\Phi_{k|k-1}P_{k-1}\Phi_{k|k-1}^{T}{H_{k}}^{T}$ and $N_{k}=C_{k}-H_{k}Q_{k}{H_{k}}^{T}-R_{k}$ Here, $C_{k}$ represents the error variance matrix, which can be expressed as

(38) $$C_{k}=\left\{\begin{array}{c} \frac{\lambda_{k-1}\varepsilon_{k}\varepsilon_{k}^{T}}{1+\lambda_{k-1}},\left(k>2\right)\\ 0.5\varepsilon_{1}\varepsilon_{1}^{T},\left(k\leq 2\right) \end{array}\right.$$

where $\varepsilon_{k}=Z_{k}-H_{k}{\hat{X}}_{k|k-1}$ denotes the estimation error. When the state changes suddenly, the increase of estimation error leads to the increase of error variance matrix and thus the increase of forgetting factor. In this way, through the introduction of forgetting factor, it can improve the tracking ability of the Kalman filter.

Secondly, we introduce the weighting parameter $d_{k}$ in the the loop iteration process of Kalman filter. Here, the weighting parameter is introduced to reduce the impact of outdated state on the current estimation and prediction, which can be calculated as

(39) $$d_{k}=\frac{1-b}{1-b^{k+1}}$$

Here, *b* can take empirical values ranging from 0.95 to 0.99. Then, the discretization matrix of the system noise covariance matrix in the k-th loop iteration of Kalman filter can be written as

(40) $$Q_{k}=\left(1-d_{k}\right)Q_{k-1}+d_{k}\left(K_{k}\varepsilon_{k}\varepsilon_{k}^{T}K_{k}^{T}+P_{k}-P_{k-1}\right)$$

and the system observation noise covariance matrix in the k-th loop iteration of Kalman filter can be written as

(41) $$R_{k}\;=\;\left(1\;-\;d_{k}\right)\;{R\;}_{k-1}\;+\;d_{k}\;\;\left(\;\left(I\;-\;\;H{\;K}_{k}\right)\varepsilon_{k}\varepsilon_{k}^{T}{\left(I\;-\;HK_{k}\right)}^{T}\;\;+\;HP_{\;k}H^{T}\right)$$

Through the above two improvements, the extended Kalman filter established based on the above “current” statistical model not only improves the ability to track state changes, but also can adaptively estimate the noise parameters by using the measured output data in the filtering process. In this way, there is no need to estimate and adjust the noise parameters manually in the practical application process, and it can also improve the performance of tracking and prediction.

Through introducing the adaptive extended Kalman filter algorithm into the SDN-FANET segmented hybrid routing mechanism, the SDN controller can track and predict the motion of UAV more accurately. Therefore, to assist the SDN controller to calculate the route, the proposed algorithm is executed on the SDN controller. Specifically, after the SDN controller receives the location information carried in the Hello packet, it will save this information. When the historical location information is enough, the SDN controller will utilize the adaptive extended Kalman filter for prediction. It is worth noting that the step size by performing of Kalman filter prediction *T* should be the update interval of the Hello packet. However, the large update interval of Hello packet can result in large estimation error. For this problem, we propose to utilize the location, velocity, and acceleration, i.e., $x,y,z,v_{x},v_{y},v_{z},a_{x},a_{y},a_{z}$ in the set of estimation state variables ${\hat{X}}_{k}$, with which we can obtain accurate location of each node. The predicted node location can be expressed as

(42) $$\left[\begin{array}{c} \hat{x}\\ \hat{y}\\ \hat{z} \end{array}\right]=\left[\begin{array}{c} x\\ y\\ z \end{array}\right]+\left[\begin{array}{c} v_{x}\\ v_{y}\\ v_{z} \end{array}\right]\Delta T+\left[\begin{array}{c} a_{x}\\ a_{y}\\ a_{z} \end{array}\right]\frac{\Delta T^{2}}{2}$$

where ΔT represents the interval between the moment of receipt of the route computation request and the last Hello packet received from the same node. After updating the node position through prediction, the SDN controller then calculates the optimal path and delivers the latest segmented routing table to the source node.

## 5. Simulations and Analysis

In this section, we conduct OPNET-software-based simulation to verify the performance of the proposed SDN-FANET hybrid routing mechanism. The simulation parameters are provided in Table 1. Two metrics are used to evaluate the performance of the proposed algorithm. The first one is end-to-end delay (EED), which is the time that a packet takes to reach the destination node from the source node. Small and stable EED means excellent real-time performance of data transmission in the network. The EED $T_{\mathrm{EED}}$ can be expressed as

(43) TEED=∑iTr−Ts∑iTr−TsNN

where $T_{s}$ represents the timestamp at which the packet is sent, $T_{r}$ represents the timestamp at which the packet is received, and *N* denotes the number of packets received at the current instant. The second one is packet delivery ratio (PDR), which can reflect the reliability of data transmission in the network. The PDR $P_{\mathrm{PDR}}$ can be calculated as

(44) PPDR=NrNrNsNs

where $N_{s}$ represents the total number of packets sent and $N_{r}$ represents the total number of received valid packets.

In the following, to verify the necessity and advantage of the introduction of SDN in FANET, both end-to-end delay and packet delivery ratio of the proposed SDN based routing scheme and classical non-SND routing schemes, e.g., DSDV and 3D-GPSR, are compared firstly. Then, in order to verify the performance of the adaptive extended Kalman prediction, performance comparisons between SDN routing without prediction and SDN routing with the proposed adaptive extended Kalman prediction are also provided. Finally, we also compare the performance of SDN routing scheme with the proposed adaptive extended Kalman prediction at different speed of UAV node, which can reflect different dynamic scenarios of FANET. In all figures, the definition of *y*-Axes is labeled at the top of the figure and the definition of *x*-Axes is simulation time for all figures.

### 5.1. Simulation Model Based on OPNET

The SDN-FANET simulation model based on OPNET includes multiple different levels of models, e.g., network layer, node layer, and process layer. The network layer model uses nodes to construct a topology that reflects the realistic network structure. In the simulations, two type of nodes, i.e., SDN controller and UAV nodes, are included in the network layer model. The location of SDN controller node is fixed. While multiple UAV nodes are mobile, constituting the FANET network. Besides, this model also includes the Rxgroup Config module and Mobility Config module. Here, the Rxgroup Config module is used to configure communication parameters such as communication radius and path loss of UAV nodes, while the Mobility Config module is used to configure mobility model and movement speed of UAV nodes.

**Figure 13:**
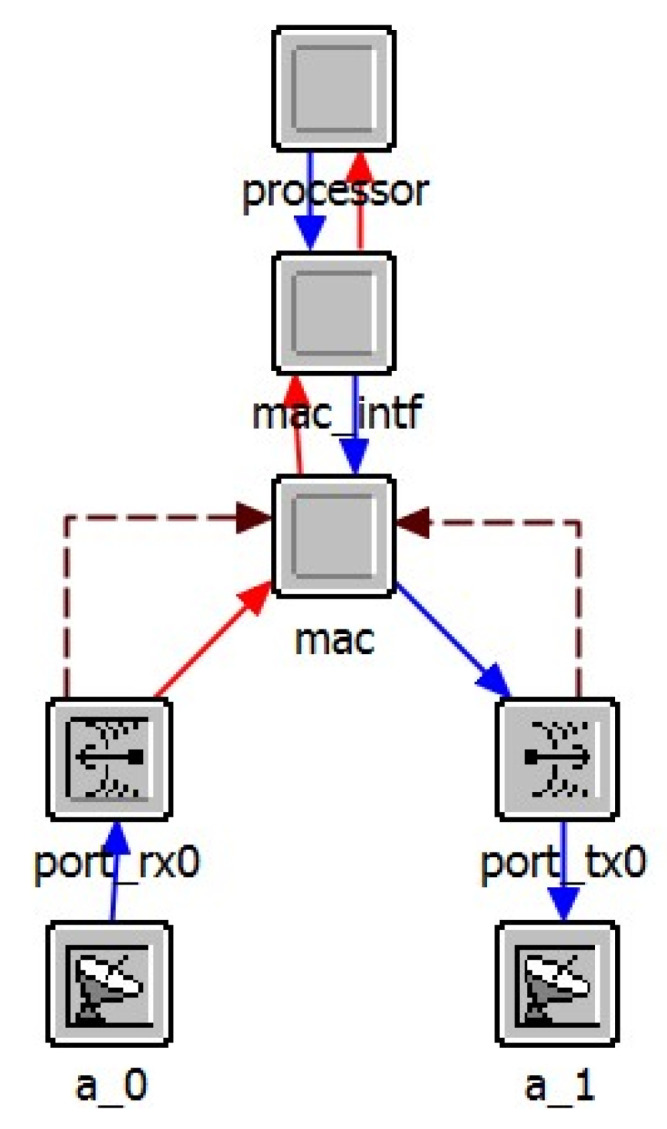
Node layer model of UAV.

**Figure 14:**
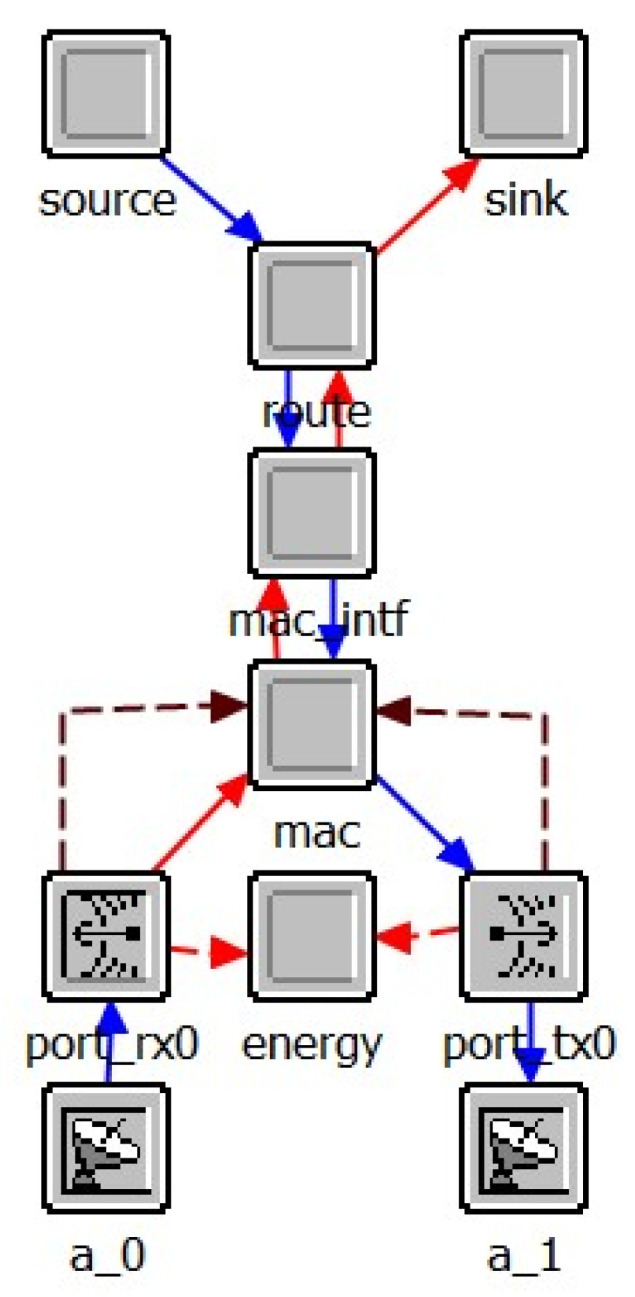
Node layer model of SDN controller.

The node layer model is used to simulate the internal functions of the network layer nodes, which combines a variety of functional modules. As shown in Figure 13, the node layer model of UAV simulates the functions of different levels of the UAV communication system. Here, the source module and the sink module belong to the application layer and simulate the generation and destruction of service data. The route module belongs to the network layer and is used to simulate various routing protocols at the network layer. The MAC module simulates the carrier sense multiple access with collision avoidance (CSMA/CA). Moreover, mac_intf module is used to simulate the interface between the Media Access Control (MAC) layer and the higher layer to implement the address resolution protocol. In the physical layer, port_tx0 and port_rx0 simulate the wireless transmitter and receiver, respectively, while a_0 and a_1 model the transmit and receive antennas, respectively. The energy module is used to model the communication energy consumption of UAV nodes. The node layer model of SDN controller is presented in Figure 14, the physical layer and MAC of which are the same as that of UAV node in general. The layer above MAC layer is the core processor module of the controller, which is used to simulate the calculation of the route and the processing and delivery of various control packets by the SDN controller.

**Figure 15:**
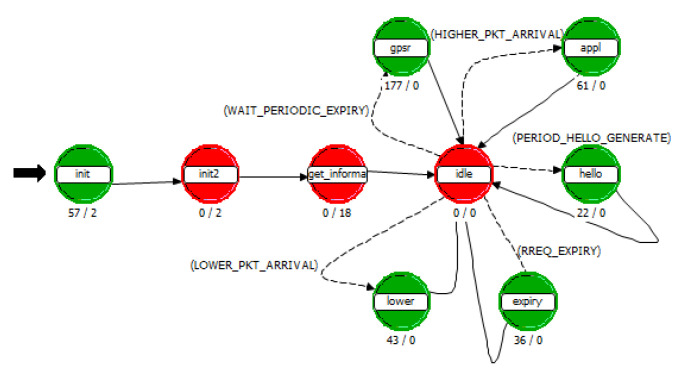
Route module process in UAV.

**Figure 16:**
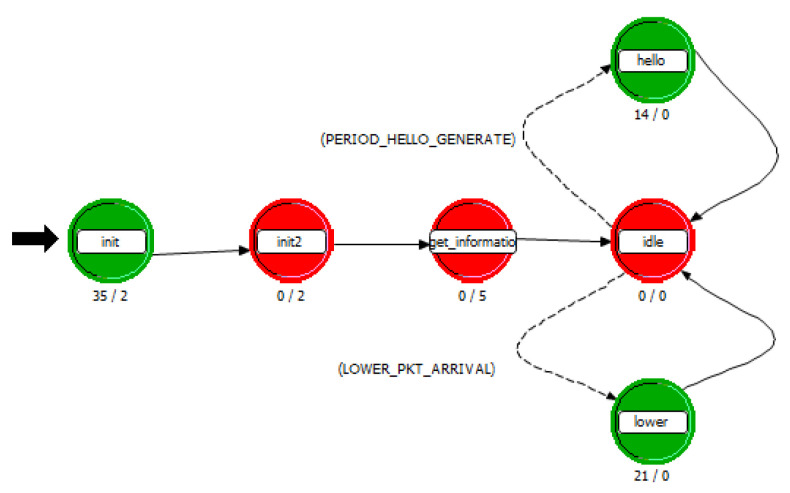
Route module process in SDN controller.

The process layer model is a concrete entity that implements various network protocol algorithms and packet processing flows. The implementation of different algorithms is realized through the pattern programming of finite state machine, utilizing the Proto-C language of OPNET Modeler. In this paper, two main process models are written to conduct the simulation of the routing algorithm. One is the route module process in the UAV node, as shown in Figure 15; the other one is the processor module process in the SDN controller node, as shown in Figure 16.

### 5.2. Simulation Analyses

**Figure 17:**
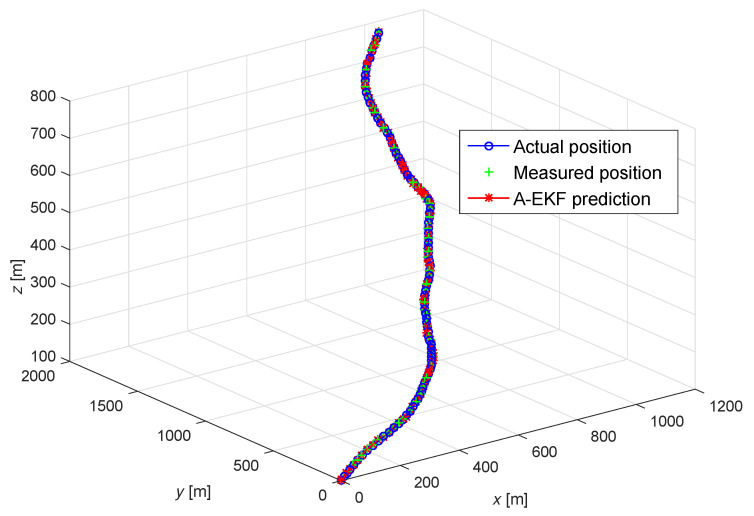
Adaptive extended Kalman prediction for position of UAV node.

**Figure 18:**
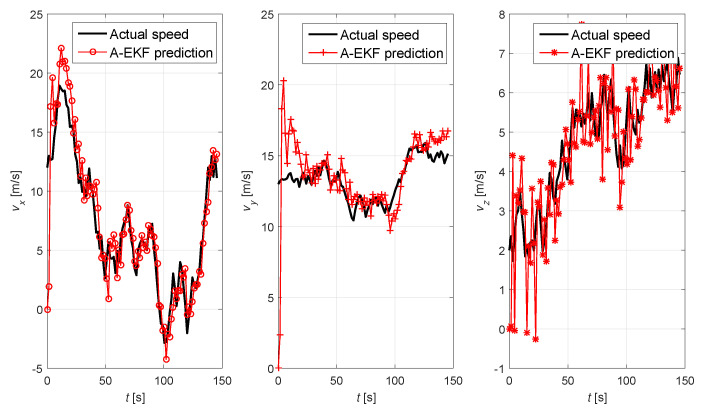
Adaptive extended Kalman prediction for speed of UAV node.

In order to verify the performance of the adaptive extended Kalman prediction, numerical simulations are carried out in MATLAB (R2023b), where the 3D Gaussian motion model is used to simulate the effect of tracking and prediction. The acceleration along the three axes follows a uniform distribution, and the initial velocities along the three axes are 4 m/s, 3 m/s, and 0 m/s, respectively. The sampling interval and measurement noise variance of the simulation are 3 s and 0.3 m/s, respectively. The results of the adaptive extended Kalman prediction of UAV motion are shown in Figure 17 and Figure 18. From Figure 17, it can be seen that the predicted results and the measured results are generally consistent with actual motion trajectory of UAV nodes, indicating that the adaptive extended Kalman prediction algorithm can accurately predict the position of the UAV nodes. Figure 18 shows that the adaptive extended Kalman algorithm can also accurately predict the velocity of the UAV node in the three directions through predicting and tracking the change of the velocity of the UAV quickly.

**Figure 19:**
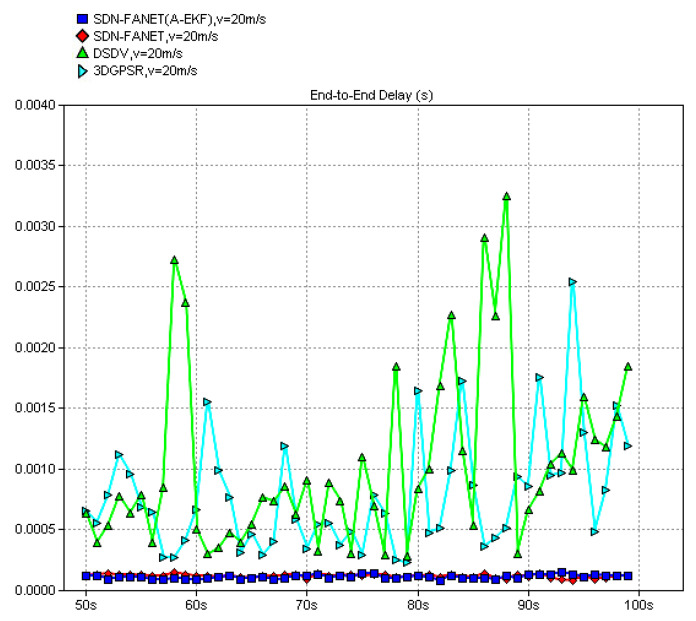
End-to-end delay of different routing schemes (v = 20 m/s).

**Figure 20:**
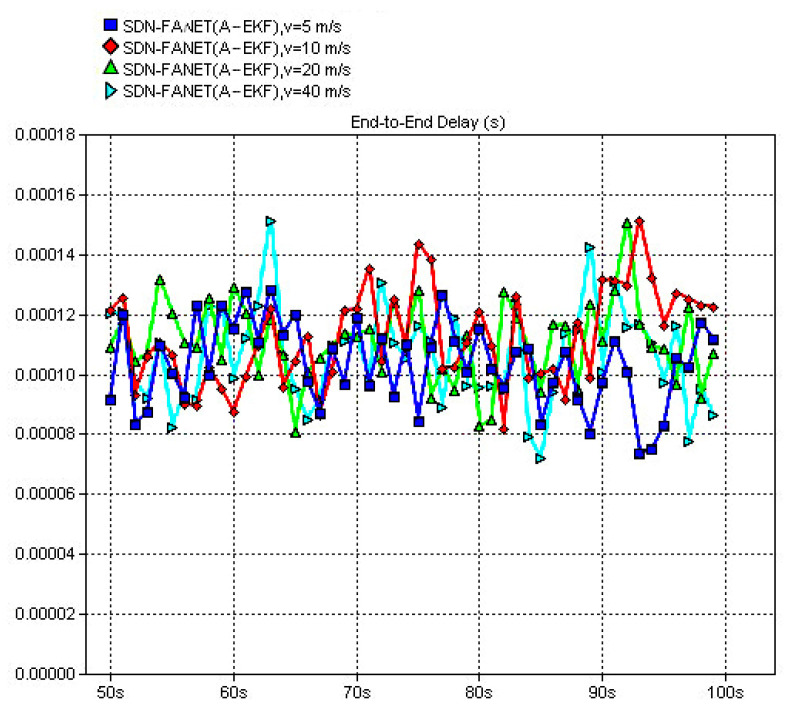
End-to-end delay of the proposed routing scheme at different speeds.

In Figure 19, we first evaluate the end-to-end delay of different routing schemes, consisting of the classical DSDV and 3DGPSR routing algorithms and the proposed routing algorithms. While SDN-FANET refers to SDN FANET segmented hybrid routing without adaptive extended Kalman prediction, SDN-FANET (A-EKF) adopts adaptive extended Kalman prediction. It can be observed from Figure 19 that the end-to-end delay of DSDV is slightly higher than that of 3DGPSR and both these algorithms have severe delay jitter. While the proposed SDN-FANET hybrid routing mechanism stabilizes at a small end-to-end delay, i.e., around 1 ms. This is because the DSDV achieves lower end-to-end delay through maintaining the routing table with extra control overhead. However, it is difficult to update the routing table quickly and effectively in high-speed motion scenes. Further, since the 3DGPSR routing is a greedy routing scheme based on geographical location, it has fast response in high-speed motion scenes, whereas the unavoidable routing holes will cause the increase of end-to-end delay, while the proposed SDN-FANET hybrid routing mechanism can better adapt to the FANET environment and obtain lower delay. Figure 20 presents the end-to-end delay of the SDN FANET segmented hybrid routing with adaptive extended Kalman prediction at different speeds of UAV nodes. It can be seen that as the speed increases, the amplitude of delay jitter becomes larger and the stability of the scheme decreases, while the proposed SDN FANET hybrid routing can maintains small average end-to-end delay.

**Figure 21:**
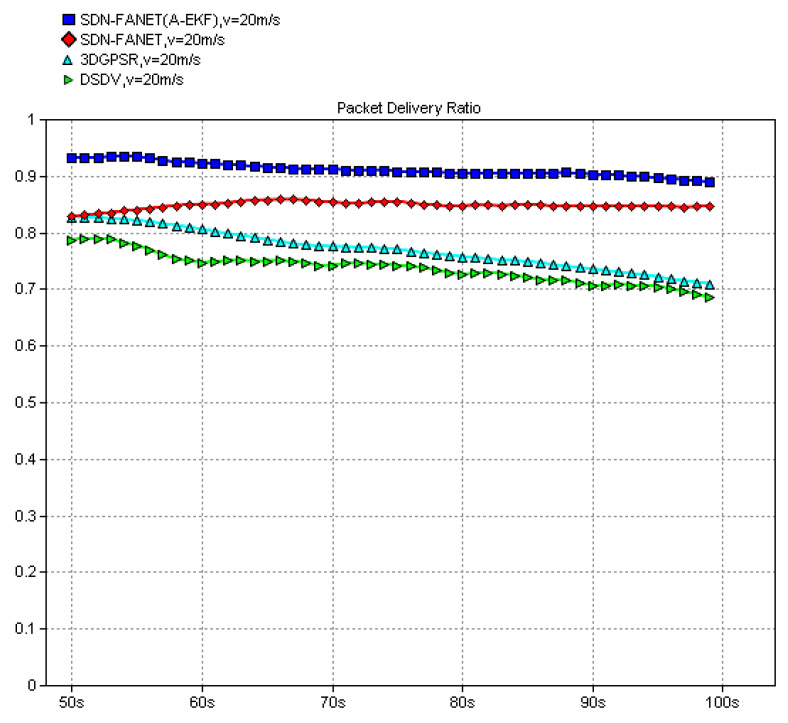
Packet delivery ratio of different routing schemes (v = 20 m/s).

**Figure 22:**
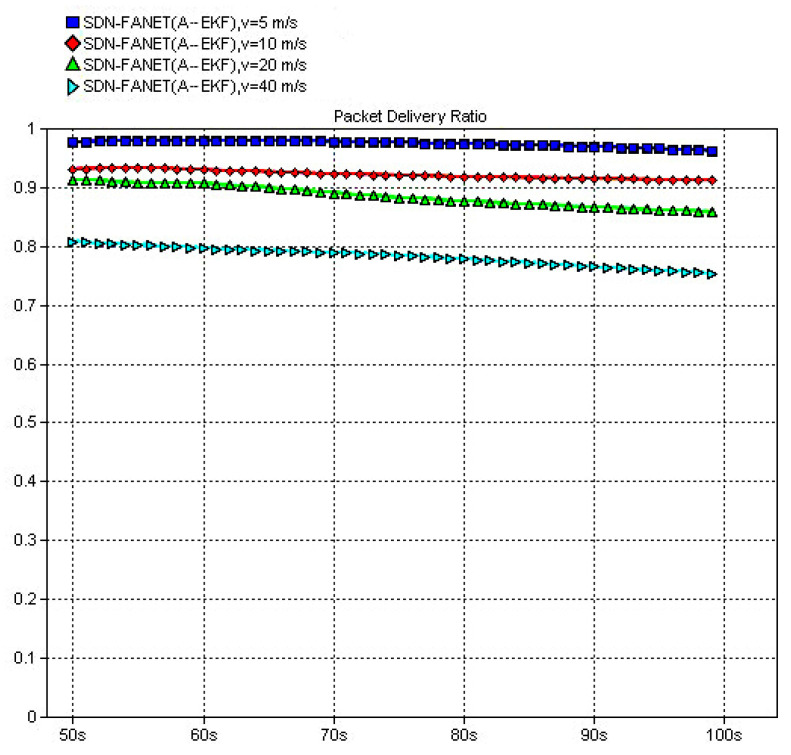
Packet delivery ratio of the proposed routing scheme at different speed.

In the following, we evaluate the packet delivery ratio. It can be observed from Figure 21 that the proposed SDN-FANET hybrid routing mechanism outperforms the DSDV, and the 3DGPSR routing and the packet delivery ratio of the SDN-FANET hybrid routing with adaptive extended Kalman prediction is over 90 percent. Figure 22 provides the packet delivery ratio of SDN-FANET hybrid routing mechanism at different speeds of UAV nodes. It can be seen from Figure 22 that the packet delivery ratio of the SDN-FANET hybrid routing with adaptive extended Kalman prediction decreases as the speed of UAV nodes increases. When the speed of UAV nodes is up to 40 m/s, the packet delivery ratio still reaches more than more than 75 percent.

## 6. Conclusions

In this paper, we have proposed a FANET segmented routing based on the hybrid SDN architecture. A unified network information update and maintenance method has been designed for the UAVs and the controller. Then, using the data forwarding method based on segmented source routing, an SDN-FANET hybrid routing has been designed. Moreover, an adaptive extended Kalman prediction algorithm has also been proposed to reduce the packet loss rate. The SDN controller has used the predicted results to accurately calculate the forwarding path. Simulation results based on OPNET have shown that the proposed routing scheme can achieve a higher packet delivery ratio and a more stable end-to-end delay performance than original solutions. However, we mainly focused on the FANET routing algorithm design under the architecture of SDN; the implementation of SDN FANET is also a critical and meaningful topic for practical application, which is left as our future work.

## Figures and Tables

**Table 1:** Simulation parameters.

Parameter	Value
Simulation area	5000 × 5000 × 2000 m
Number of UAV nodes	30
Coverage radius of UAV node	1200 m
Mobility model of UAV node	Random Waypoint
Carrier frequency	2.4 GHz
Transmission rate	54 Mbps
Packet delivery rate	5 packets/s
Packet size	1024 bits
Initial energy of UAV node	40 w
Simulation time	100 s
MAC protocol	IEEE 802.11 g
Update cycle of Hello packet	3 s

## Data Availability

Data are contained within the article.

## References

[1] Agrawal J., Arafat M.Y. (2025). Bio-Inspired Algorithms for Efficient Clustering and Routing in Flying Ad Hoc Networks. Sensors.

[2] Chen J., Xu Y., Wu Q., Zhang Y., Chen X., Qi N. (2019). Interference-aware online distributed channel selection for multicluster FANET: A potential game approach. IEEE Trans. Veh. Technol..

[3] Erkalkan E., Topuz V., Buldu A. (2024). Addressing the Return Visit Challenge in Autonomous Flying Ad Hoc Networks Linked to a Central Station. Sensors.

[4] Nazib R.A., Moh S. (2020). Routing protocols for unmanned aerial vehicle-aided vehicular ad hoc networks: A Survey. IEEE Access.

[5] Rozario A., Ahmed E., Mansoor N. (2024). A Robust Routing Protocol in Cognitive Unmanned Aerial Vehicular Networks. Sensors.

[6] Khan M.F., Yau K.L.A., Noor R.M., Imran M.A. (2019). Routing schemes in FANETs: A survey. Sensors.

[7] Yuan Y., Ren G., Cai X., Li X. (2024). An Adaptive 3D Neighbor Discovery and Tracking Algorithm in Battlefield Flying Ad Hoc Networks with Directional Antennas. Sensors.

[8] Khan I.U., Qureshi I.M., Aziz M.A., Cheema T.A., Shah S.B.H. (2020). Smart IoT control-based nature inspired energy efficient routing protocol for flying ad hoc network (FANET). IEEE Access.

[9] Al-Emadi S., Al-Mohannadi A. Towards enhancement of network communication architectures and routing protocols for FANETs: A Survey. Proceedings of the 2020 3rd International Conference on Advanced Communication Technologies and Networking (CommNet).

[10] Oubbati O.S., Atiquzzaman M., Lorenz P., Tareque M.H., Hossain M.S. (2019). Routing in flying ad hoc networks: Survey, constraints, and future challenge perspectives. IEEE Access.

[11] Wang H., Li Y., Zhang Y., Huang T., Jiang Y. (2023). Arithmetic Optimization AOMDV Routing Protocol for FANETs. Sensors.

[12] Hussen H.R., Choi S.C., Park J.H., Kim J. Performance analysis of MANET routing protocols for UAV communications. Proceedings of the 2018 Tenth International Conference on Ubiquitous and Future Networks (ICUFN).

[13] Tan X., Zuo Z., Su S., Guo X., Sun X., Jiang D. (2020). Performance analysis of routing protocols for UAV communication networks. IEEE Access.

[14] Choi S.C., Hussen H.R., Park J.H., Kim J. Geolocation-based routing protocol for flying ad hoc networks (FANETs). Proceedings of the 2018 Tenth International Conference on Ubiquitous and Future Networks (ICUFN).

[15] Ronzani D., Bujari A., Palazzi C.E. A hybrid reactive and position-based approach to packet routing in 3D topology networks. Proceedings of the 2018 Wireless Days (WD).

[16] Wang F., Chen Z., Zhang J., Zhou C., Yue W. Greedy forwarding and limited flooding based routing protocol for UAV flying ad-hoc networks. Proceedings of the 2019 IEEE 9th International Conference on Electronics Information and Emergency Communication (ICEIEC).

[17] Fu J., Cui B., Wang N., Liu X. (2019). A distributed position-based routing algorithm in 3D wireless industrial internet of things. IEEE Trans. Ind. Inform..

[18] Yuan Z., Huang X., Sun L., Jin J. Software defined mobile sensor network for micro UAV swarm. Proceedings of the 2016 IEEE International Conference on Control and Robotics Engineering (ICCRE).

[19] Kreutz D., Ramos F.M.V., Veríssimo P.E., Rothenberg C.E. (2015). Azodolmolky, Siamak and Uhlig, Steve. Software-Defined Networking: A Comprehensive Survey. Proc. IEEE.

[20] Qu H., Xu X., Zhao J., Yue P. An SDN-based space-air-ground integrated network architecture and controller deployment strategy. Proceedings of the 2020 IEEE 3rd International Conference on Computer and Communication Engineering Technology (CCET).

[21] Sami Oubbati O., Atiquzzaman M., Ahamed Ahanger T., Ibrahim A. (2020). Softwarization of UAV Networks: A Survey of Applications and Future Trends. IEEE Access.

[22] Barritt B., Kichkaylo T., Mandke K., Zalcman A., Lin V. Operating a UAV mesh internet backhaul network using temporospatial SDN. Proceedings of the IEEE Aerospace Conference.

[23] Zhang X., Wang H., Zhao H. An SDN framework for UAV backbone network towards knowledge centric networking. Proceedings of the IEEE INFOCOM 2018–IEEE Conference on Computer Communications Workshops (INFOCOM WKSHPS).

[24] Singhal C., Rahul K. LB-UAVnet: Load balancing algorithm for UAV based network using SDN. Proceedings of the 2019 22nd International Symposium on Wireless Personal Multimedia Communications (WPMC).

[25] Guerber C., Larrieu N., Royer M. Software defined network based architecture to improve security in a swarm of drones. Proceedings of the 2019 International Conference on Unmanned Aircraft Systems (ICUAS).

[26] Zhao L., Saldin A., Hu J., Fu L., Shi J., Guan Y. A novel simulated annealing based routing algorithm in F-SDNs. Proceedings of the IEEE INFOCOM 2020–IEEE Conference on Computer Communications Workshops (INFOCOM WKSHPS).

[27] Deshpande A.A., Chiariotti F., Zanella A. SMURF: Reliable multipath routing in flying ad-hoc networks. Proceedings of the 2020 Mediterranean Communication and Computer Networking Conference (MedComNet).

[28] Abdullah Z.N., Ahmad I., Hussain I. (2019). Segment routing in software defined networks: A Survey. IEEE Commun. Surv. Tutor..

[29] Ventre P.L., Salsano S., Polverini M., Cianfrani A., Abdelsalam A., Filsfils C., Camarillo P., Clad F. (2021). Segment routing: A comprehensive survey of research activities, standardization efforts, and implementation results. IEEE Commun. Surv. Tutor..

[30] Poularakis K., Qin Q., Marcus K.M., Chan K.S., Leung K.K., Tassiulas L. Hybrid SDN Control in Mobile Ad Hoc Networks. Proceedings of the 2019 IEEE International Conference on Smart Computing (SMARTCOMP).

[31] Wang J., Miao Y., Zhou P., Hossain M.S., Rahman S.M.M. (2016). A software defined network Routing in Wireless Multihop Network. J. Netw. Comput. Appl..

[32] Gankhuyag G., Shrestha A.P., Yoo S. (2017). Robust and reliable predictive routing strategy for flying ad-hoc networks. IEEE Access.

[33] Lin L., Sun Q., Li J., Yang F. (2012). A novel geographic position mobility oriented routing strategy for UAVs. J. Comput. Inf. Syst..

[34] Li X., Huang J. ABPP: An adaptive beacon scheme for geographic routing in FANET. Proceedings of the 2017 18th International Conference on Parallel and Distributed Computing, Applications and Technologies (PDCAT).

[35] Liu C., Zhang G., Guo W., He R. (2020). Kalman prediction-based neighbor discovery and its effect on routing protocol in vehicular ad hoc networks. IEEE Trans. Intell. Transp. Syst..

[36] Rovira-Sugranes A., Razi A. Predictive routing for dynamic UAV networks. Proceedings of the 2017 IEEE International Conference on Wireless for Space and Extreme Environments (WiSEE).

[37] Song M., Liu J., Yang S. A mobility prediction and delay prediction routing protocol for UAV networks. Proceedings of the 2018 10th International Conference on Wireless Communications and Signal Processing (WCSP).

